# Modeling the significance of corporate social responsibility on green capabilities and sustainability performance

**DOI:** 10.1016/j.heliyon.2024.e38991

**Published:** 2024-10-05

**Authors:** Han Xiao, Abdullah Al Mamun, Mohammad Nurul Hassan Reza, Xiaofang Lin, Qing Yang

**Affiliations:** a School of Business, Nantong Institute of Technology, Nantong City, Jiangsu Province, 226000, China; b UKM - Graduate School of Business, Universiti Kebangsaan Malaysia, 43600, UKM Bangi, Selangor Darul Ehsan, Malaysia; c Faculty of Business and Management, UCSI University, Taman Connaught, 56000, Cheras, Kuala Lumpur, Malaysia

**Keywords:** Corporate social responsibility, Green dynamic capabilities, Green innovation capabilities, Sustainable performance

## Abstract

This study examines the complex interplay among corporate social responsibility (CSR), green dynamic capabilities, green innovation capabilities, and organizations' economic, environmental, and social performance in the current era of innovation and sustainability. The proposed framework was tested using data from 634 medium- and large-sized Chinese manufacturing firms. Partial least squares structural equation modeling was employed to analyze the data. The findings demonstrate that CSR to external and internal stakeholders positively influences green dynamic capabilities. Meanwhile, CSR to external stakeholders and green dynamic capabilities are positively associated with green innovation capabilities, subsequently influencing the economic, environmental, and social performance of manufacturing firms. Moreover, the results reveal the mediating effect of green dynamic capabilities on the association between CSR and green innovation capabilities. Green innovation capabilities also mediate the association between CSR to external stakeholders and green dynamic capabilities on manufacturing organizations’ economic, environmental, and social performance. This study offers a novel exploration of the intricate interplay among CSR, green dynamic capabilities, green innovation capabilities, and organizational performance within manufacturing organizations. These findings provide valuable insights for managers and offer opportunities for further research that will ultimately contribute to a deeper understanding of the roles of sustainability and innovation in modern corporations.

## 1 Introduction

Climate change and environmental pollution have become central to the societal discourse. The world is facing major environmental, social, and humanitarian challenges, sparking the need for an adequate response from organizations [1]. Consequently, consumers and governments stress serious concerns and demand that firms practice alternative stances toward business sustainability [2,3]. Business sustainability encompasses three dimensions: environmental, social, and financial [4]. It has become extremely important for organizations to focus on their overall business sustainability, not just financial performance [5].

Manufacturing firms, particularly in the face of increasing pressure from stakeholders to enhance their nonfinancial performance [6], are witnessing a growing credibility of corporate social responsibility (CSR), especially within the manufacturing sector [7,8]. Even the concept of CSR has become an inescapable component of firms. CSR can be defined as the notion that firms are responsible for the societies they serve, the environment from which they draw their resources, and their profit activities [9]. CSR involves engaging with both external and internal stakeholders. Internal stakeholders are individuals or groups that have an interest or concern in an organization's operations and are affected by its activities [10]. Examples of internal stakeholders include customers, employees, and suppliers. External stakeholders are affected by the organization's actions, such as the natural environment, future generations, and human species [9]. Examples of external stakeholders include regulatory bodies, non-government organizations (NGOs) and industry associations. Understanding the expectations and influences of these diverse stakeholders is crucial for implementing effective CSR strategies that can enhance firm performance. Despite the wealth of research on CSR and firm performance, there is a notable gap in the literature regarding the synergistic effects of green dynamic capabilities (GDC) and green innovation capabilities (GIC) on this relationship, particularly in the Chinese context. GDC is defined as an organization's ability to integrate, build, and reconfigure internal and external competencies to address rapidly changing environmental conditions [11]. This encompasses the capacity to innovate in response to environmental challenges, including the development of new products, processes, and business models that are environmentally sustainable. GIC refers to a firm's ability to develop new products or processes that contribute to its environmental sustainability [12]. These include innovations that reduce environmental harm, enhance resource efficiency, or promote the use of renewable resources. Thus, shifting to green innovation has become one way to achieve holistic prosperity. Hao and He [13] suggested that green innovation is a critical strategy for firms to achieve sustainable financial and environmental performance.

The assertion of CSR in sustainable business practices has attracted attention from the global scientific community. As Chen et al. [14] established, firms are compelled to adopt green innovation due to internal and external pressures. These practices, in turn, contribute to the enhancement of business sustainability. Aguinis and Glavas [15] provided a comprehensive literature review of the CSR literature and highlighted key areas where further research is needed. It discusses how CSR can lead to various organizational outcomes, including performance improvements. Le [16] demonstrated evidence that CSR disclosure laws positively impact green innovation and contribute to sustainable firm performance. Consequently, a direct and positive relationship between CSR and the adoption of green practices and corporate sustainability is evident in existing literature [17]. Unfortunately, the same conclusion has yet to be reached, as evidence produced by other authors creates a conspiracy. For example, Anser, Zhang and Kanwal [18] identified the importance of CSR practices in organizational performance. While Becker [19] highlighted, little is known about whether green innovation affects organizational performances compared to those that do not adopt. Additionally, studies have shown that CSR practices can significantly promote green product and process innovation, which in turn can enhance firm performance [20]. Moreover, GDCs have been identified as a mediator between CSR and green innovation, suggesting that the development of such capabilities is vital for translating CSR efforts into tangible innovative outcomes. Additionally, the role of green innovation as a mediator between CSR and financial performance highlight the complex nature of these dynamics [21]. Contradictions in the literature, such as the finding that CSR does not directly affect firm performance but does so through mediating factors such as green transformational leadership and green innovation, suggest that the direct and indirect effects of CSR on firm performance are multifaceted and require further exploration [22].

China, as the world's second-largest economy and a major industrial powerhouse, faces unique challenges and opportunities in this arena [23]. The Chinese government has been actively promoting green initiatives and sustainable development through stringent regulations and policies [24]. Nonetheless, Chinese firms often grapple with balancing economic performance with environmental and social responsibilities. This dichotomy presents a fertile ground for investigating how green practices influence overall firm performance within this context. Ervits [25] drew attention to the incongruity in reaching a consensus on CSR and firm performance in China. The extant literature has explored the sustainability performance of firms globally. However, there is a lack of literature offering a perspective on the business sustainability performance of manufacturing firms in China [4]. Nonetheless, over the past few decades, China has emerged as a predominant economic power with a significant impact on the global economy. As Chinese organizations expand their international reach, they are confronted with the complex task of balancing economic growth with environmental conservation and social welfare. In response, an increasing number of Chinese firms have adopted CSR practices, recognizing their obligation to contribute to a more sustainable future.

The extant literature has not fully elucidated the intricate relationships between CSR practices, stakeholder influence, GDC, GIC, and sustainable performance in the context of medium and large Chinese firms. Medium and large firms in China are defined by a set of criteria that includes the number of employees and product sales revenue. Medium-sized enterprises are those with the number of employees exceeding 300 to less than 1000 and product sales revenue exceeding RMB 20 million to less than 400-million-yuan. Large enterprises, on the other hand, are defined as those with 1000 or more employees and product sales revenue exceeding RMB 400 million [26]. These classifications are significant, as they reflect the scale of operations and the potential impact of these enterprises on the national economy. The standards have been jointly studied and formulated by various state commissions and bureaus, ensuring that they are tailored to China's economic structure and development goals [27,28]. These organizations face distinct challenges and opportunities that require a more detailed examination. Considering the inconclusive findings and scarcity of research on the relationship between CSR practices, green innovation, and business sustainability, particularly in the Chinese context, this study aims to investigate this correlation. Investigating the dynamics of CSR, GDCs, GIC, and firm performance is essential because of the increasing importance of sustainable development in the business environment. The interplay between these factors is crucial for organizations, especially in emerging markets, to achieve a balance between economic success and environmental stewardship [20,21]. Thus, it is of utmost importance to conduct an investigation into this matter because China is a global manufacturing hub and holds a significant share of the global economy [29]. Specifically, this study addressed the following research questions.

RQ1 Do the CSR practices of the manufacturing firms influence their GDC and GIC?

RQ2 Is GDC positively associated with GIC?

RQ3 Is GIC positively associated with sustainability performance?

RQ4 Do GDC and GIC mediate the relationship between CSR practices and sustainable performance?

RQ5 Do firm size, age, origin, and cross-border e-commerce engagement influence the associations between variables?

To answer these research questions, this study surveys 634 Chinese medium and large manufacturing firms and investigates the impact of CSR on GDC and GIC toward sustainable performance by integrating GDC and GIC as mediators. By incorporating the dynamic capability theory, this study expands our understanding of how organizations adapt to external pressures and innovate to meet stakeholder demands. This study carries great importance, as it adds to the existing body of literature on CSR, sustainability, and stakeholder management, specifically within the unique context of China. The novelty of the study lies in its comprehensive examination of the interplay between CSR, GDC, GIC, and sustainable performance across economic, environmental, and dimensions. The integration of “three green” elements—green strategy, green CSR, and green innovation—into a single model provides a comprehensive framework for examining the impact on sustainable firm performance. Interestingly, while most studies have focused on the direct impact of CSR on firm performance, this study contributes to the literature by highlighting the mediating role of GDC and GIC in this relationship. It suggests that CSR practices not only directly promote green products and process innovation but also enhance a firm's dynamic capabilities, which in turn drive sustainable performance. Thus, this study provides valuable practical insights for organizations seeking to enhance their sustainability practices, innovate responsibly, and align their CSR strategies with stakeholders' diverse expectations in a constantly evolving global environment. While the focus is on Chinese firms, the implications of this study extend far beyond China. Understanding how green capabilities and CSR initiatives can drive performance is critical for businesses worldwide, especially as global markets increasingly prioritize sustainability. The findings can inform best practices for integrating CSR with green capabilities in various cultural and regulatory contexts, helping firms worldwide to enhance their sustainability efforts. Policymakers in other countries can draw lessons from China's approach to promoting green initiatives and devising strategies to encourage sustainable business practices within their jurisdictions. Global organizations can leverage the insights from this study to GDC and GIC, providing a competitive edge in the increasingly sustainability-focused global market.

The remainder of this paper is organized as follows. Section 2 provides the literature review and hypotheses development. Section 3 describes the research methodology. Section 4 presents the results of the current study. Section 5 discusses these results, including their theoretical contributions, managerial implications, limitations, and future research directions. Finally, Section 6 concludes the paper.

## 2 Literature review

### 2.1 Dynamic capability view

The dynamic capability view, a fundamental and influential perspective in the broader field of strategic management, serves as the theoretical foundation for this study. This theory offers a robust framework for understanding how organizations, particularly medium and large Chinese firms, navigate external pressures, innovate, and adapt to achieve sustainable performance. Teece, Pisano and Shuen [30] developed the dynamic capability view and posited that dynamic capabilities are essential to an organization's ability to adapt and succeed in rapidly changing environments. At the core of the dynamic capability view is the concept of dynamic capabilities, encompassing an organization's ability to integrate, build, and reconfigure its internal and external competencies in response to dynamic environments. Dynamic capabilities, as opposed to static routines or processes, are the driving force behind an organization's ability to evolve and adapt over time [31].

One of the key premises of this study is that CSR practices are of utmost importance to an organization's ability to adapt and respond to the changing expectations of its stakeholders. CSR encompasses a range of activities aimed at balancing an organization's economic, environmental, and social responsibilities. It equips organizations with the capacity to acquire new competencies, reconfigure existing ones, and innovate in response to societal and environmental shifts [18]. In this context, CSR practices can be viewed as dynamic capabilities that foster GIC and sustainability [9].

Furthermore, stakeholders, including external and internal, play a crucial role in shaping an organization's CSR practices [10]. The dynamic capability view theory posits that stakeholder influence is an external driver that stimulates organizations to develop new capabilities and competencies. Stakeholder pressures, including demands for transparency, ethical behavior, and environmental responsibility, act as catalysts for change and innovation [11].

In this study, dynamic capabilities serve as the mechanism through which stakeholder influence is translated into organizational capabilities that enable organizations to identify and seize new opportunities, respond to changing regulations, and develop innovative strategies to promote sustainability [32]. Li [33] points out that GDC acts as a link between CSR practices, green product development, and competitive advantage, leading to better organizational performance. Simultaneously, innovations driven by CSR initiatives not only enhance a company's economic performance but also promote improved environmental and social outcomes, benefiting all three aspects of the triple bottom line [9]. Therefore, this study posits that GDC and GIC can mediate the relationship between CSR and an organization's sustainable performance. As organizations develop dynamic capabilities, they are better equipped to achieve sustainable performance across the economic, environmental, and social dimensions [9]. By incorporating the dynamic capability view theory, this study acknowledges that innovation, particularly GIC, is vital to CSR practices. Green innovation involves the development and implementation of novel products, processes, and technologies to minimize environmental harm [34]. It manifests dynamic capabilities at work as organizations adapt to environmental pressures by innovating responsibly. The theoretical underpinnings of this study acknowledge sustainability as a comprehensive concept that transcends environmental issues. It encompasses economic feasibility, environmental accountability, and social welfare [35]. The dynamic capability view theory is complementary in that it posits that dynamic capabilities enable organizations to respond to the multifaceted aspects of sustainability, including the economic, environmental, and social dimensions [34].

The dynamic capability view theory is the cornerstone of this study's theoretical framework. It offers a comprehensive approach to understanding how CSR practices influenced by stakeholders contribute to developing dynamic capabilities that, in turn, lead to innovation and sustainable performance. By incorporating this theory in the context of medium and large Chinese firms, this study enhances our understanding of how organizations adapt, innovate, and achieve sustainability in a rapidly changing global business environment. This theoretical foundation informs empirical investigations and illuminates the complex relationships that underpin sustainability in a dynamic and stakeholder-driven world.

### 2.2 CSR to external stakeholders and GDC

External stakeholders include not only regulatory bodies and industry associations but also NGOs. CSR to such stakeholders and organizations is significant when the lack of consideration given by the firms toward sustainability initiatives [36]. Organizations actively engaged in CSR initiatives aimed at external stakeholders are more likely to establish a robust foundation for developing GDC [9]. These CSR practices contribute to human capital development, ensure legal compliance with environmental matters, and foster a culture of adaptability. Industrialization is a major driver of economic growth. However, it exploits natural resources, causing global warming and other environmental issues. Firms need to innovate their business activities in an eco-friendly way, which helps reduce energy and natural resource consumption as well as waste production [37]. In addition, Martínez‐Ros and Kunapatarawong [38] demonstrated that the perception of environmental protection is related to GDC, as the dynamic capacity of the firms is the incorporation and reconfiguration of external and internal resources towards environmental protection.

Further, engaging with NGOs as external stakeholders can provide valuable insights, partnerships, and resources related to environmental sustainability efforts. This alignment and cultural shift within the organization enhances its ability to sense and respond to environmental changes, thereby facilitating the development of GDC. Hence, we propose the following hypothesis.

H1 *A positive association exists between CSR to external stakeholders and green dynamic capabilities.*

### 2.3 CSR to internal stakeholders and GDC

CSR directed at internal stakeholders primarily focuses on initiatives benefiting them. Internal stakeholders encompass employees, customers and suppliers. The expectations of internal stakeholders motivate and encourage organizations to voluntarily develop and renew their resources and capabilities to align with current environmental requirements [10]. On the other hand, GDC involves the capacity to identify green opportunities, develop green processes and products, and continually adjust to changing environmental conditions. Organizations must be prepared to modify and replenish their internal and external resources because of the potential loss of support from stakeholders if they are not transparent about environmental and social issues [39].

Hence, organizations must develop GDC to deepen their relationships with stakeholders. Organizations that actively engage in CSR initiatives targeting internal stakeholders, including societal well-being, environmental protection, sustainability, and employee participation, are more likely to build a robust foundation for GDC development [11]. These CSR practices contribute to resource accumulation, foster stakeholder engagement and collaboration, strategically align social responsibility with environmental sustainability, and promote learning and adaptation. This alignment and cultural shift enhance an organization's ability to sense and respond to environmental changes, thereby facilitating the development of GDC [40]. Hence, this discussion leads to the following hypothesis.

H2 *A positive relationship exists between CSR to internal stakeholders and green dynamic capabilities*.

### 2.4 CSR to external stakeholders and GIC

CSR to external stakeholders can be classified into two categories: primary and secondary stakeholders. Primary stakeholders include the natural environment, future generations, and nonhuman species. Secondary stakeholders encompass environmental interest groups and animal welfare group organizations [41]. In addition to expectations from external stakeholders, organizations face pressure from external stakeholders to adapt their business practices and align themselves with environmental challenges. For example, environmental violations, such as water contamination, can create normative pressure on organizations from NGOs that focus on environmental welfare [42]. NGOs, which are external stakeholders, can also affect unsustainable business practices through their environmental reports, which may lead to a boycott of unsustainable products.

Consequently, various factors motivate organizations to adopt environmentally sustainable practices in production of their products and services. Li et al. [43] noted that organizations voluntarily align with environmental challenges, responding to expectations from both external and internal stakeholders. Watson et al. [44] posited that external stakeholders compel organizations to innovate and reconcile their operational techniques. Additionally, organizations actively implementing CSR practices aimed at external stakeholders are more likely to cultivate GIC, which involves the adoption of new skills, techniques, procedures, and approaches to environmentally sustainable innovation [20]. This positive relationship arises from employee empowerment and satisfaction, customer demand for green products, legal compliance driving innovative solutions, and the alignment of CSR values with environmentally sustainable innovation objectives. Thus, we propose the following hypothesis.

H3 *There is a positive relationship between CSR to external stakeholders and GIC.*

### 2.5 CSR to internal stakeholders and GIC

Internal stakeholders can significantly impact an organization's GIC. GIC involves the ability to adopt new skills, techniques, procedures, and approaches that align with environmental responsibility and contribute to long-term sustainability goals. The extant literature has established a direct relationship between CSR practices in response to internal stakeholder expectations and GIC in different contexts. Dai and Lu [45] conducted an empirical study on 201 manufacturing firms, and their findings revealed that responding to public opinion by innovating is associated with the ability of organizations to consider environmental protection.

Hu et al. [46] made an intriguing discovery — government intervention aimed at promoting eco-innovation through subsidies and favorable policies can lead organizations to use their resources to produce innovative products that produce less pollution. The demand from consumers for products that have fewer negative impacts on the environment results in firms using their adapted resources to create eco-friendly products in response to market demand [47]. Hence, the changing consumer dynamics drive firms to develop GIC [48]. Borah et al. [49] noted that the environmental effects of firms can be attributed to their utilization of resources, waste produced, and the discharge of waste produced. Zhang, Zhang [29] posited that the government's disclosure of a firm's environmental actions significantly impacts green innovation. These practices align organizational values with environmentally sustainable innovation, encourage forward-thinking investments in green solutions, foster employee engagement and the introduction of fresh perspectives, and facilitate collaboration with environmentally conscious consumers and other internal stackholders. Such alignment, engagement, and partnerships enhance an organization's ability to innovate in ways that are both green and sustainable. Hence, the associated hypothesis was as follows.

H4 *There is a significant relationship between CSR to internal stakeholders and GIC.*

### 2.6 GDC and GIC

GDC is the higher-level capability of an organization to establish green and sustainable development in a constantly changing market [50]. Organizations not only innovate by utilizing existing resources but also create new knowledge by utilizing these resources [51]. Specifically, GDC enables organizations to adapt swiftly to evolving environmental regulations, market demands for sustainable products, and emerging green technologies. This adaptability extends to fostering a culture of environmental awareness and green innovation [52].

Consequently, the positive and significant impact of GDC on GIC of an organization is achieved through the integration, reallocation, and construction of internal and external resources, which facilitate the production of environmentally sustainable products [53]. In addition, GDC helps organizations achieve sustainability and green development in a rapidly changing business environment [34]. As a result, organizations must possess the necessary capabilities and resources, including advanced technology, sustainable processes, and employee expertise, to significantly reduce environmental pollution and resource consumption while achieving GIC. GDC extends the principles of dynamic capabilities to achieve specific green and sustainable objectives, allowing organizations to manage sustainability-related risks and thrive in green innovation. Thus, we propose the following hypothesis.

H5 *There is a positive relationship between GDC and GIC*.

### 2.7 GIC and economic (ECP), social (SOP), and environmental performance (ENP)

An organization's ECP encompasses various metrics, including return on investment, profits, and sales, and is typically characterized by a formal tone. Previous investigations have demonstrated that eco-friendly innovation has a favorable influence on an organization's expenses, which ultimately contributes to its financial performance [54]. The implementation of green innovation strategies can lead to the creation of unique products and services and enhance the reputation of organizations in the market, thereby facilitating the recovery of the costs associated with these techniques [55]. Green innovations set organizations apart from their competitors, driving increased market share, revenue growth, and enhanced profitability and return on investment [49].

Moreover, they help organizations mitigate costs, achieve regulatory compliance, reduce environmental risks, and potentially improve stock prices. Fan and Wang [56] examined the performance of 19 companies. The results confirm that the implementation of green innovation is a competitive advantage that promotes the financial performance of environmentally conscious companies. Mubeen, Nisar [57] illustrated valuable insights for SME practitioners, emphasizing the importance of incorporating green innovation and value co-creation in business strategies to boost sustainable performance. Aftab et al. [58] shed light on the interplay between ethical considerations, innovation, and organizational performance in the context of environmental sustainability. Miroshnychenko, Barontini and Testa [59] also proved that an organization's implementation of green practices significantly impacts its financial performance. Marín-Vinuesa et al. [60] concluded that investment in green innovation resources can positively impact an organization's financial performance. As organizations increasingly prioritize green innovation, their economic performance reaps the benefits of sustainable practices and products, aligning environmental responsibility with financial success. Thus, the relevant hypothesis was as follows.

H6 *There is a positive relationship between GIC and ECP.*

Social performance encompasses improvements in stakeholder welfare, safety, and community health. It also includes protecting employees and minimizing risk to the public [61]. The literature has widely acknowledged that green innovation is positively associated with organizational social performance. By actively engaging in green innovation, organizations contribute to community well-being and development, support charitable and philanthropic causes, invest in employee development and welfare, foster positive relationships with local communities, and establish a positive reputation among stakeholders [62]. The implementation of environmentally sustainable practices by organizations positively impacts employee morale and customer relationships, thereby enhancing the brand's reputation. Kanan et al. [63] provided guidelines for policymakers in manufacturing firms on how to use green human resource management practices to strengthen employees' commitment to the environment, ultimately maximizing organizations' social performance.

Shen et al. [64] discovered that, in addition to an organization's environmental and economic performance, its social performance is also positively impacted. As organizations prioritize green innovation, they not only benefit the environment but also actively participate in and promote social welfare, exemplifying the interconnectedness of sustainability efforts with positive social performance [65]. Thus, we propose the following hypothesis.

H7 *There is a positive association between GIC and SOP.*

Environmental performance encompasses the strategic efforts undertaken by organizations to surpass societal expectations regarding the environment [53]**.** Researchers have demonstrated that the environmental performance of organizations depends significantly on their GIC. For example, Oliva et al. [66] posited that the superior quality of environmentally friendly products and services, the processes used to produce them, and the integration of ecological concerns into the product development process contributed to positive environmental performance. Kratzer, Meissner and Roud [67] suggested that green innovation should be considered a proactive stance in the realm of environmental product development aimed at improving the environmental performance of products and services in a highly competitive market. Organizations with strong GIC actively reduce their carbon footprints, implement waste reduction programs, decrease the emissions of hazardous substances, and continuously monitor and improve their environmental impacts [68]. Such organizations recognize their commitment to environmental sustainability and comply with all relevant environmental regulations and standards. Rehman et al. [69] showed that promoting GIC through strategic human resource practices can enhance environmental sustainability within manufacturing firms. Similarly, Adegbile, Sarpong and Meissner [70] demonstrated that green innovation is associated with organizational management practices, leading to enhanced environmental performance. Organizations significantly improve their environmental performance by adopting new skills and production techniques and committing to sustainable innovation [71]. Green innovation aligns organizations with best sustainability practices, resulting in a reduced environmental footprint and commitment to environmental responsibility, which is reflected in their overall environmental performance. Thus, the associated hypothesis was as follows.

H8 *There is a positive relationship between GIC and ENP.*

### 2.8 The mediating role of GDC and GIC

It is common for organizations that adopt CSR practices to allocate resources towards sustainability initiatives such as green technology, renewable energy, and waste reduction. Such CSR practices can cultivate a culture of innovation and continuous learning within an organization, thereby fostering an innovation mindset that aligns with the dynamic capabilities necessary to address evolving environmental challenges [33]. This is because a firm's ability to reinvent resources correlates positively with its performance in producing environmentally sustainable products [10]. Li [33] highlighted that GDC serves as a bridge between CSR practices, green product development, and competitive advantage, thereby improving organizational performance. Therefore, this study views GDC as a mediator in the relationship between CSR, GIC, and sustainable performance. In addition, previous research indicates that GIC implementation improves firms' financial [60], environmental [68] and social performance [61].

Furthermore, it has been established that GDC can lead to GIC. Organizations that effectively implement CSR-driven innovations are often recognized as market leaders in sustainability. This leadership translates into a competitive advantage as consumers and stakeholders increasingly prioritize products and services that reflect responsible and sustainable practices [60]. The innovations spurred by CSR initiatives not only enhance a company's economic performance but also contribute to improved environmental and social outcomes, thereby positively impacting all three pillars of the triple bottom line [9]. Implementing GICs empowers firms to achieve long-term and sustainable competitive advantage by continually developing and integrating green innovations, ensuring that their sustainable performance remains consistent and robust over time. Thus, the associated hypotheses were as follows.

H9 *GDC mediates the relationship between CSR to external stakeholders and GIC*

H_10-12_. *GIC mediate the relationship between CSR to external stakeholders with ECP, SOP, and ENP*.

H13 *GDC mediates the relationship between CSR to internal stakeholders and GIC*

**H**_**14-16**_*GIC mediate the relationship between CSR to internal stakeholders with ECP, SOP, and ENP*.

**H**_**17-19**_. *GIC mediates the relationship between GDC with ECP, SOP, and ENP*.

**Fig. 1:**
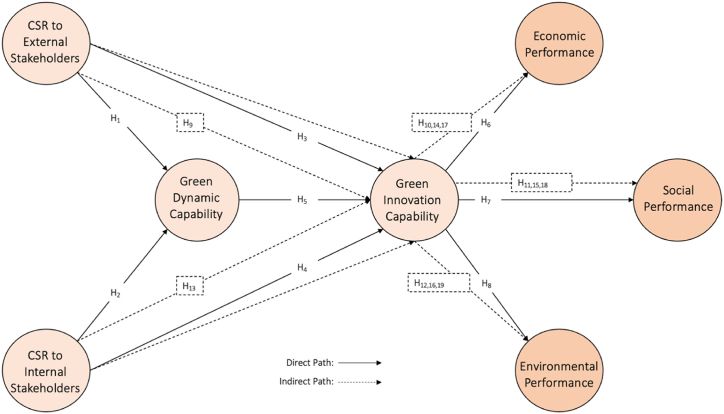
Research framework.

All associations hypothesized above are presented in Fig. 1.

## 3 Research methodology

### 3.1 Research design

The primary purpose of this study was to examine the impact of CSR practices on GI and SP in medium and large firms. To accomplish this, we adopted a quantitative approach using a cross-sectional design. Accordingly, a self-administered electronic questionnaire was administered to participants. We used a non-random sampling technique, specifically convenience sampling, to select the sample for this study. Convenience sampling enables researchers to meet certain criteria such as convenience of access, geographic proximity, availability, and consent to participate. Similarly, Dai, Siddik and Tian [72] used convenience sampling to examine the impact of CSR on environmental performance. While convenience sampling offers practical advantages it also presents notable weaknesses. These include a lack of generalizability, as the sample may not be representative of the broader population, and potential selection bias, since participants are chosen based on their availability and willingness to participate. Despite these limitations, the use of convenience sampling is justified in this study due to the practical constraints of time and resources, as well as the exploratory nature of the study.

### 3.2 Population and sample

The respondents targeted for this study were employees of medium and large Chinese manufacturing firms in Jiangsu Province. This includes a blend of local enterprises deeply rooted in the nation's culture and international companies drawn to the thriving Chinese market. Middle and senior management employees including executive director/CEO, executive or senior management and middle management were selected because of their access to and insight into organizational strategies that were essential for the scope of this study. We selected firms that have actively embraced and implemented CSR. Focusing on these individuals, we aim to gain a comprehensive understanding of the experiences, insights, and perspectives of those in charge of companies that prioritize social responsibility and sustainability.

Additionally, to ensure a representative sample size, we followed the guidelines of Hair et al. [73], who suggested that the sample size should be based on the total number of predictors. Based on this criterion, we conducted a G∗Power test using effect size (*f*^2^) of 0.15, α-err prob of 0.05, power (1-β err prob) of 0.80, and four predictors. The estimated sample size initially determined by the G∗Power test was 85. However, we decided to expand our sample size beyond this initial estimate based on the recommendations of the software.

### 3.3 Survey instrument

The questionnaire consisted of two sections. The initial section encompassed participants’ demographic characteristics, including age, sex, tenure, firm origin, and firm engagement. The second section comprises seven variables adopted from the literature. CSR to external stakeholders included seven items, whereas CSR to internal stakeholders consisted of nine items. All CSR items were adapted from Turker [74], who utilized exploratory factor analysis to validate the measurement items. Six GIC and five GDC items were taken from Xue, Boadu and Xie [75] and Yuan and Cao [20], respectively. Six items of ECP, six items for SOP and five items of ENP were adapted from Aftab et al. [76]. The validity of these items was established through adequate Alpha values. Complete instrument presented in the Supporting Material S1.

The integrated variables were measured on a five-point Likert scale ranging from 1 = Strongly Disagree to 5 = Strongly Agree. A pre-test was conducted with a similar group of people before approaching the final data collection to ensure a clear understanding of the questions. To address potential language barriers for the Chinese participants, the questionnaire was translated into Mandarin and back into English with the assistance of experts. The pre-test results enabled the researchers to improve and modify certain questions, ultimately leading to the final distribution of the questionnaire.

### 3.4 Data collection method

Ethical approval was obtained from the research ethics committee to ensure the ethical safety and credibility of the research. We implemented several measures to ensure that only middle and senior management employees filled out the online questionnaire. First, we collaborated with the human resources (HR) departments of the participating organizations to exclusively identify and reach out to individuals holding middle and senior management positions. The HR department directly sent the survey invitations to the intended respondents. Second, the invitation email and introductory section of the online questionnaire explicitly stated that the survey was intended solely for middle and senior management employees. This clarification aimed to deter ineligible employees from participating. Additionally, the questionnaire included a screening question at the beginning, asking respondents to confirm their roles within the organization. Only those who identified themselves as middle or senior managers were allowed to participate in the survey. Responses from individuals who did not meet this criterion were excluded from the data set. Additionally, to ensure the validity of the results, the researchers took precautionary measures to exclude incomplete responses. Two criteria were established to identify such responses: answers that were the same for all items and responses submitted within less than 5 min. After excluding six questionnaires, 634 participants were included in the data analysis, meeting the minimum sample size requirement of 146 participants. The data collection period spanned from December 2022 to February 2023.

### 3.5 Common method bias

The researchers took necessary precautions to address the potential issue of common method bias before conducting the data analysis. This bias occurs when the same method is used to measure constructs, leading to specific correlations between independent and dependent variables [77]. To avoid this issue, we administered Harman's single-factor test, which yielded a single factor of 28.31 %. The results indicated that there was no common method bias as a single factor explained less than 50 % of the variance [78]. In addition, we conducted a full collinearity test, as shown in (Table 1), which confirmed that each construct's variance inflation factor was less than 3.3, as suggested by Kock, Berbekova and Assaf [79]. The results revealed that the data were free from common method bias.

**Table 1:** Full collinearity test.

	CES	CIS	GDC	GIC	ECP	SOP	ENP
Variance Inflation Factors	1.403	1.601	2.006	2.044	1.532	1.677	1.456

**Note:** CES - CSR to External Stakeholders; CIS - CSR to Internal Stakeholders; GDC - Green Dynamic Capability; GIC - Green Innovation Capability; ECP - Economic Performance; SOP - Social Performance; ENP - Environmental Performance.

### 3.6 Multivariate normality

This study employed a web-based statistical tool to examine multivariate normality and to calculate skewness and kurtosis. The results showed that the p values of skewness and kurtosis were less than 0.05, which confirms that the distribution is not normal.

### 3.7 Data analysis method

Because of the non-normality issue, this study employed partial least squares structural equation modeling (PLS-SEM), specifically the SmartPLS software, to analyze the data. This technique is well-suited for predictive modeling and can effectively examine the relationships between multiple constructs and indicators, allowing us to assess the predictive capabilities of the theoretical framework [80]. This study involved a complex structural model with a multitude of constructs, indicators, and interrelated model relationships, making PLS-SEM an efficient tool for handling these intricacies [81]. Additionally, this study aligns with the spirit of exploratory research aimed at theory development. PLS-SEM is a powerful tool in this regard, enabling the exploration of theoretical extensions, facilitating the development of new perspectives, and enhancing the understanding of the research domain [82].

## 4 Summary of findings

### 4.1 Demographics

Table 2 presents the demographic information of the sample, which comprised 634 observations. Of these, 47.6 % were male, and 52.4 % were female. Regarding the age group, the largest group (40.9 %) was within the 36–45 age range, followed by those aged 26–35 years (29.5 %). Respondents aged 46–55 (23.8 %) represented those in their late career stages and possibly in senior management roles. A smaller portion (4.7 %) was aged 56–65, and a limited segment (1.1 %) was over 65. Notably, no respondents were 18–25 years old, possibly implying a participant pool with more professional experience and maturity, thus providing valuable insights into the study's age diversity. With respect to tenure, the majority of respondents (50.0 %) had 6–10 years of experience. A substantial proportion (19.9 %) fell within the 1–5-year category, followed by those aged 11–15 (18.1 %).

**Table 2:** Demographic characteristics.

	N	%		N	%
*Gender*			*Education*		
Male	302	47.6	Diploma/Advanced Diploma	91	14.4
Female	332	52.4	Bachelor degree or equivalent	333	52.5
Total	634	100.0	Master Degree or equivalent	118	18.6
			PhD or DBA	92	14.5
*Age Group*			Total	634	100.0
18–25 years	0	0			
26–35 years	187	29.5	*Position*		
36–45 years	259	40.9	Executive Director/CEO	163	25.7
46–55 years	151	23.8	Executive or senior management	341	53.8
56–65 years	30	4.7	Middle management	130	20.5
More than 65 years	7	1.1	Total	634	100.0
Total	634	100.0			
			*Firm Established*		
*Tenure*			Less than 1 year	6	0.9
Less than 1 year	23	3.6	1–5 years	31	4.9
1–5 years	126	19.9	6–10 years	163	25.7
6–10 years	317	50.0	11–15 years	245	38.6
11–15 years	115	18.1	16–20 years	123	19.4
16–20 years	32	5.0	More than 20 years	66	10.4
More than 20 years	21	3.3	Total	634	100.0
Total	634	100.0			
			*Type of Firm*		
*Firm Origin*			Textiles manufacturing	95	15.0
Chinese (Local)	251	39.6	Petroleum, chemicals and plastics	102	16.1
Chinese (Multinational)	275	43.4	Electronics equipment	96	15.1
International	108	17.0	Food production	45	7.1
Total	634	100.0	Metal manufacturing	49	7.7
			Wood, leather and paper	70	11.0
*Cross-Border eCommerce Engagement*			Pharmaceutical products	35	6.0
None	62	9.8	Non-metallic mineral products	20	3.2
≤20 %	161	25.4	Automobile manufacturing	54	8.5
21 %–30 %	228	36.0	Transportation	35	5.5
31 %–40 %	109	17.2	Waste resources	12	1.9
41 %–50 %	32	5.0	Other manufacturing	18	2.8
More than 50 %	42	6.6	Total	634	100.0
Total	634	100.0			
			*Firm Size*		
			Medium Enterprise	465	73.3
			Large Enterprise	169	26.7
			Total	634	100.0

The survey also included professionals with job tenures of 16–20 years (5.0 %) and more than 20 years (3.3 %), reflecting the experience of seasoned professionals. A small minority (3.6 %) of respondents had less than one year of job experience. Regarding the origins of the firms, 43.4 % of the participants were affiliated with Chinese multinational corporations, 39.6 % with local Chinese enterprises, and 17.0 % with international organizations. The Chinese manufacturing sector is dominated by local and multinational Chinese firms, with a smaller proportion of international companies. The distribution in our sample reflects this characteristic of the population, reinforcing the representativeness of our sample. According to the data, the level of cross-border e-commerce engagement varies significantly, with approximately 36.0 % of individuals participating at a rate of 21–30 %, 25.4 % at 20 % or lower, and 17.2 % at 31–40 %. These findings suggest a diverse range of involvement in international online commerce. In terms of education, more than half (52.5 %) of the respondents had a bachelor's degree or equivalent, followed by a master's degree or equivalent (18.6 %). A diverse range of organizational positions was represented among the survey participants, with 53.8 % holding executive or senior management positions, 25.7 % in executive director CEO roles, and 20.5 % in middle management.

Of the responding firms, 73.3 % were medium-sized, while large enterprises accounted for 26.7 %. According to recent statistics from the National Bureau of Statistics of China (https://data.stats.gov.cn/english/), the distribution of medium and large enterprises in the Chinese manufacturing sector shows a higher proportion of medium enterprises compared to large enterprises, which aligns with our sample distribution. Among the respondents, 38.6 % were from firms established–11-15 years, 25.7 % from firms established–6-10 years, and 19.4 % from firms established–16-20 years, highlighting diverse organizational development stages. The breakdown of firm type shows a varied assortment of industries, with petroleum, chemicals, and plastic manufacturing accounting for 16.1 %, textile manufacturing for 15.0 %, and electronics equipment manufacturing for 15.1 %. Additionally, there was notable representation from the wood, leather, and paper (11.0 %) and automobile manufacturing (8.5 %) sectors. The manufacturing sector in China is highly diverse, with significant representation across various sub-industries. The proportions of firms in our sample from key industries such as textiles, electronics, and chemicals are consistent with the broader industry distribution in China.

### 4.2 Measurement model

**Fig. 2:**
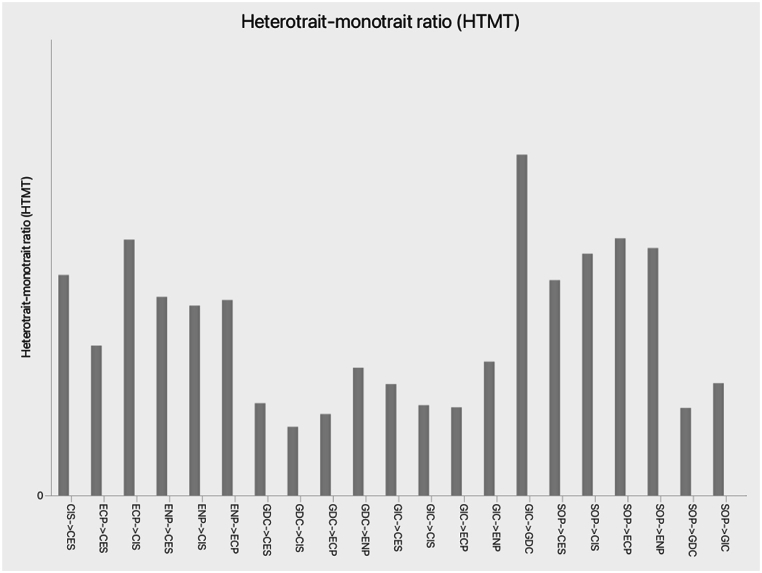
Heterotrait-monotrait ratio (HTMT) Matrix. **Note:** CES - CSR to External Stakeholders; CIS - CSR to Internal Stakeholders; GDC - Green Dynamic Capability; GIC - Green Innovation Capability; ECP - Economic Performance; SOP - Social Performance; ENP - Environmental Performance.

Table 3 presents the reliability and validity of the constructs. Based on these results, the values of Cronbach's alpha and composite reliability (rho_a, rho_c) of all constructs were higher than the recommended threshold of 0.7 [82]. In addition, the convergent validity of the constructs was achieved, as the values of average variance extracted (AVE) were higher than the cut-off of 0.5. As Hair, Matthews [82] suggest, a latent variable possesses adequate convergent validity if its AVE value is higher than 0.5. Moreover, Henseler, Hubona and Ray [83] recommended that if the heterotrait-monotrait ratio (HTMT) value is below 0.85, discriminant validity is achieved. The results of the HTMT (Fig. 2) confirm that discriminant validity is established as the HTMT values of all the construct values are less than 0.85 [83]. Furthermore, the results of the Fornell-Larcker criterion (included in Supporting Material S2) show that the square roots of AVE are higher than the internal correlations and coefficients in each row and column, indicating that discriminant validity was achieved following the guidance of [83].

**Table 3:** Reliability and validity.

Variables	No. Items	Mean	Standard Deviation	Cronbach's Alpha	Composite reliability (rho_a)	Composite reliability (rho_c)	Average Variance Extracted	Variance Inflation Factors
CES	7	5.539	1.137	0.911	0.940	0.929	0.650	1.263
CIS	9	5.349	1.077	0.906	0.922	0.922	0.569	1.243
GDC	5	5.009	1.637	0.948	0.949	0.960	0.828	1.046
GIC	6	4.566	1.601	0.932	0.934	0.946	0.747	1.000
ECP	6	5.106	1.212	0.874	0.885	0.904	0.611	–
SOP	6	5.188	1.144	0.875	0.886	0.905	0.615	–
ENP	5	5.365	1.153	0.873	0.881	0.907	0.662	–

**Note:** CES - CSR to External Stakeholders; CIS - CSR to Internal Stakeholders; GDC - Green Dynamic Capability; GIC - Green Innovation Capability; ECP - Economic Performance; SOP - Social Performance; ENP - Environmental Performance.

### 4.3 Structural model

**Fig. 3:**
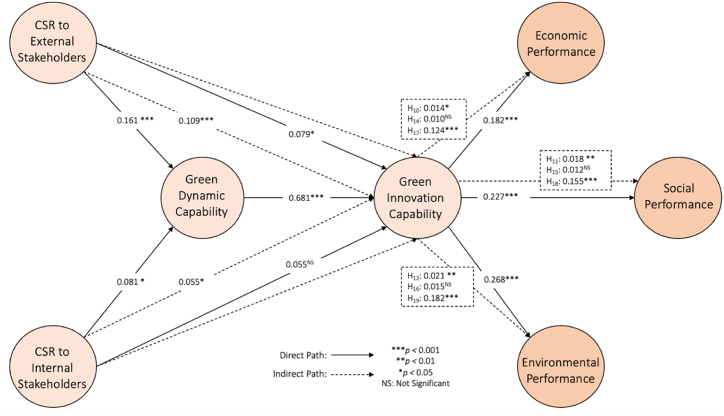
Measurement model.

To obtain the results of hypothesis testing, we conducted a bootstrapping analysis using SmartPLS. The results presented in Fig. 3 and Table 4 indicate that seven of the eight direct relationships are statistically significant. The findings showed that CSR to external stakeholders (β = 0.161, t = 3.620, p = 0.000) had a statistically significant impact on GDC. Hence, H_1_ is supported. Similarly, the results confirmed a positive and significant (β = 0.081, t = 1.804, p = 0.036) association between CSR to internal stakeholders and GDC. Thus, H_2_ is supported.

**Table 4:** Hypothesis testing.

Hypothesis		Beta	CI Min	CI Max	*t* Value	*p* Value	Decision
H_1_	CES → GDC	0.161	0.090	0.237	3.620	0.000	Supported
H_2_	CIS → GDC	0.081	0.013	0.160	1.804	0.036	Supported
H_3_	CES → GIC	0.079	0.026	0.137	2.288	0.011	Supported
H_4_	CIS → GIC	0.055	−0.001	0.113	1.595	0.055	Not Supported
H_5_	GDC → GIC	0.681	0.630	0.727	23.278	0.000	Supported
H_6_	GIC → ECP	0.182	0.123	0.257	4.433	0.000	Supported
H_7_	GIC → SOP	0.227	0.165	0.299	5.556	0.000	Supported
H_8_	GIC → ENP	0.268	0.205	0.339	6.566	0.000	Supported
*Mediating Effects*							
H_9_ CES → GDC → GIC		0.109	0.061	0.160	3.655	0.000	Supported
H_10_ CES → GIC → ECP		0.014	0.004	0.029	1.891	0.029	Supported
H_11_ CES → GIC → SOP		0.018	0.005	0.035	1.937	0.026	Supported
H_12_ CES → GIC → ENP		0.021	0.006	0.041	2.005	0.023	Supported
H_13_ CIS → GDC → GIC		0.055	0.009	0.108	1.815	0.035	Supported
H_14_ CIS → GIC → ECP		0.010	0.000	0.025	1.303	0.096	Not Supported
H_15_ CIS → GIC → SOP		0.012	0.000	0.029	1.389	0.083	Not Supported
H_16_ CIS → GIC → ENP		0.015	0.000	0.032	1.475	0.070	Not Supported
H_17_ GDC → GIC → ECP		0.124	0.083	0.174	4.500	0.000	Supported
H_18_ GDC → GIC → SOP		0.155	0.112	0.203	5.670	0.000	Supported
H_19_ GDC → GIC → ENP		0.182	0.139	0.231	6.499	0.000	Supported

**Note:** CES - CSR to External Stakeholders; CIS - CSR to Internal Stakeholders; GDC - Green Dynamic Capability; GIC - Green Innovation Capability; ECP - Economic Performance; SOP - Social Performance; ENP - Environmental Performance.

Surprisingly, the results of CSR to internal stakeholders on GIC were found to be statistically insignificant (β = 0.055, t = 1.595, p = 0.055). Therefore, H_4_ was rejected. However, CSR to external stakeholders was established as a positive and significant predictor of GIC (β = 0.079, t = 2.288, p = 0.011). Therefore, H_3_ is supported. Similarly, H_5_ is supported by the results, which showed a positive and significant relationship between GDC and GIC (β = 0.681, t = 23.278, p = 0.000). The results also established a significant relationship between GIC and ECP (β = 0.182, t = 4.443, p = 0.000), thereby supporting H_6_. Furthermore, the association between GIC and SOP was found to be statistically positive and significant (β = 0.227, t = 5.556, p = 0.000), thus supporting H_7_. Finally, the results indicated a significant and positive relationship between GIC and ENP (β = 0.268, t = 6.566, p = 0.000), supporting H_8_.

Additionally, we evaluated specific indirect effects (mediating relationships). The results are presented in Table 4. The results showed that GDC mediates the relationships between CSR to external stakeholders and GIC (β = 0.109, t = 3.655, p = 0.000), CSR to internal stakeholders, and GIC (β = 0.055, t = 1.815, p = 0.035); thereby, supporting H_9_ and H_13_. Additionally, the mediating effect of GIC between CSR to external stakeholders and ECP (β = 0.014; t = 1.891; p = 0.029), CSR to external stakeholders and SOP (β = 0.018; t = 1.937; p = 0.026), CSR to external stakeholders and ENP (β = 0.021; t = 2.005; p = 0.023), were found to be positive and significant, thus, supporting H_10-12_. Surprisingly, the findings showed that GIC does not mediate relationship between CSR to internal stakeholders and ECP (β = 0.010; t = 1.303; p = 0.096) CSR to internal stakeholders and SOP (β = 0.012; t = 1.389; p = 0.083) CSR to internal stakeholders and ENP (β = 0.015; t = 1.475; p = 0.070), therefore, rejecting H_14-16_. However, GIC mediates the relationship between GDC and ECP (β = 0.124, t = 4.500, p = 0.000), GDC and SOP (β = 0.155, t = 5.670, p = 0.000), GDC and ENP (β = 0.182, t = 6.499, p = 0.000), thus, supporting H_17-19_.

In addition, we evaluated the effect size (*f*^2^), which is a measure of the extent to which exogenous factors substantially influence endogenous variables based on unique variance rather than shared variance [84]. Cohen (2013) classified effect sizes into the following categories: trivial (<0.02), minor (≥0.02), medium (≥0.15), and substantial (≥0.35). Table 5 presents the results of the effect-size analyses. The results show that the effects of CSR on external and internal stakeholders in GDC and GIC are minor. However, the effect of GDC on GIC was substantial. Finally, the effects of GIC on ECP, SOP, and ENV were minor.

**Table 5:** Effect size.

Variables	GDC	GIC	ECP	SOP	ENP
CES	0.022	0.010			
CIS	0.006	0.005			
GDC		0.902			
GIC			0.034	0.054	0.077

**Note:** CES - CSR to External Stakeholders; CIS - CSR to Internal Stakeholders; GDC - Green Dynamic Capability; GIC - Green Innovation Capability; ECP - Economic Performance; SOP - Social Performance; ENP - Environmental Performance.

### 4.4 Multigroup analysis (MGA)

As presented in Table 6, findings of the PLS-SEM multigroup analyses (MGA) revealed that there was no significant difference between the two age groups of firms with respect to the relationships under examination (H_1_-H_8_). This implies that the relationships among CSR, GDC, GIC, and sustainable performance remain stable and consistent, regardless of how long a firm has been in operation. This finding implies that the mechanisms and dynamics linking these variables are resilient to the influence of firm age.

**Table 6:** Multigroup analysis.

Associations		Firm Established ≤10 Years (N = 200) >10 Years (N = 434) Difference		Firm Origin Local (N = 251) Multinational/International (N = 383) Difference	
		Beta	p-value	Beta	p-value
H_1_	CES → GDC	−0.152	0.057	0.028	0.386
H_2_	CIS → GDC	0.054	0.264	0.040	0.375
H_3_	CES → GIC	−0.047	0.276	0.106	0.079
H_4_	CIS → GIC	0.117	0.053	0.085	0.136
H_5_	GDC → GIC	0.049	0.211	−0.134	0.020
H_6_	GIC → ECP	−0.016	0.421	0.205	0.005
H_7_	GIC → SOP	−0.074	0.185	0.189	0.007
H_8_	GIC → ENP	0.108	0.101	0.061	0.216
Associations		Firm Size Medium Firms (N = 465) Large Firms (N = 169) Difference		Cross-Border eCommerce Engagement ≤30 % Sales (N = 220) >30 % Sales (N = 362) Difference	
		Beta	p-value	Beta	p-value
H_1_	CES → GDC	0.052	0.418	0.370	0.000
H_2_	CIS → GDC	0.465	0.004	0.132	0.143
H_3_	CES → GIC	0.023	0.407	0.174	0.036
H_4_	CIS → GIC	0.015	0.437	0.136	0.050
H_5_	GDC → GIC	−0.144	0.013	−0.467	0.000
H_6_	GIC → ECP	0.170	0.015	0.199	0.008
H_7_	GIC → SOP	0.217	0.004	0.332	0.000
H_8_	GIC → ENP	0.150	0.262	0.196	0.005

**Note:** CES - CSR to External Stakeholders; CIS - CSR to Internal Stakeholders; GDC - Green Dynamic Capability; GIC - Green Innovation Capability; ECP - Economic Performance; SOP - Social Performance; ENP - Environmental Performance.

In assessing the influence of firm origin (local or international), the relationships between CSR, GDC, and GIC remained consistent across both types of firms. This finding suggests that the implementation of CSR practices has a uniform impact on GDC and GIC development in firms of varying origins. However, we found significant differences in the relationships between GDC and GIC, confirming that the effect of GDC on GIC is significantly higher among international and/or multinational firms. Furthermore, findings revealed significantly higher effects of GIC on ECP and SOP among local firms compared to international and/or multinational firms. These results may have implications for the strategies adopted by international firms in local contexts. It also provides opportunities for further research on sustainability and innovation.

We found significant differences between CSR to internal stakeholders and GDC in the context of firm size (medium and large firms), confirming that the effect of CSR to internal stakeholders on GDC is significantly higher among medium-sized manufacturing firms in China. Furthermore, we observed significant differences in the relationships between GDC and GIC, indicating a significantly higher effect of GDC on GIC in large firms. Furthermore, findings revealed significantly higher effects of GIC on ECP and SOP among medium-sized manufacturing firms in China. In the context of cross-border e-commerce engagement (≤30 % sales and ≥30 % sales), we found differences in the effect of CSR to external stakeholders on GDC and GIC, the effect of CSR to internal stakeholders on GIC, and the effect of GIC on sustainability performance, confirming that the effects are significantly higher among the firms focused on local markets. However, the effect of GDC on GIC among firms focused on the international market is significantly higher. These results offer practical insights for medium and large organizations, emphasizing the multifaceted relationship between CSR, innovation, and sustainability and paving the way for future research to explore these dynamics further.

## 5 Discussion

This study examines the significance of CSR practices in green innovation and sustainability performance within medium and large manufacturing firms in China. In particular, this study assessed the impact of CSR to external and internal stakeholders on ECP, SOP, and ENP of manufacturing firms by integrating GDC and GIC as mediators. Eight direct hypotheses are proposed, seven of which are supported. In addition, of the 17 mediating hypotheses, 11 received support, while the remaining six were rejected. The following discussion presents the main findings of the study.

In terms of CSR to external stakeholders, this study established the association between CSR to external stakeholders stakeholders and GDC (H_1_), articulating that natural resources, the physical environment, and societal well-being play an important role in forming firms' GDC. These findings are consistent with the results of Amaranti et al. [85], who highlighted the importance of considering natural resources while evaluating CSR to firms' external stakeholders stakeholders and their impact on GDC.The results also reveal that the presence of CSR to internal stakeholders enhances GDC performance, confirming H_2_. This finding underscores the significant influence of stakeholders, including employees, customers, and suppliers, significantly impact a firm's capability to adapt to and innovate environmental management practices in response to changing external and internal situations. This finding is in line with prior research [11,40] that has established that CSR to internal stakeholders leads to the adaptation of firms' resources to changing market conditions due to environmental impacts.

Conversely, the result suggests that a positive and significant impact of CSR to external stakeholders on GIC (H_3_) exists, indicating that the perception of responsible usage of natural resources and the well-being of society and non-living things affect firms' inclination to innovate green products. This finding is consistent with the previous literature. Moreover, CSR to internal stakeholders was found to have an insignificant impact on GIC (H_4_), postulating that the positive influence of customers, suppliers, and government on organizations' innovative activities, when aligned with their environmental goals and values, drives GIC. The findings is inconsistent with the previous work conducted by Dogbe, Tian [48] and Borah et al. [49]. Moreover, we found that GDC significantly promotes GIC (H_5_), demonstrating that firms’ ability to adapt their resources leads to GIC. These findings were consistent with those of Zhang et al. [86].

Furthermore, this study confirms that GIC has a significant positive impact on sustainability performance. This indicates that organizations with robust GIC are better equipped to reduce costs through improved efficiency in business processes and make a significant contribution to societal well-being by reducing waste, exploiting natural resources, and investing in green technology. As a result, these firms can improve their environmental, social, and economic performance, supporting H_6-8_. These findings concur with those of previous research, which established that GIC has a positive relationship with economic [87], social [65] and environmental performance [53,68].

The positive and significant mediating role of GDC in establishing the relationships between CSR to external stakeholders and GIC (H_9_) and CSR to internal stakeholders and GIC (H_13_) shows that pressure from CSR to external and internal stakeholders leads to GIC. The results are supported by Mousavi, Bossink and Vliet [40] and Singh et al. [11], who found that CSR to external stakeholders leads to GDC, while Aboelmaged and Hashem [10] and Martínez‐Ros and Kunapatarawong [38] confirmed that GDC promotes GIC. Based on these findings, it can be inferred that stakeholder pressure can lead to GIC when the firm adapt to its current business environment. In addition, this study demonstrates that CSR to external and internal stakeholders not only has a direct relationship with GIC but also has an indirect effect on GIC through GDC. This finding is consistent with that of Yuan and Cao [20], who confirm that GDC plays a mediating role in the context of CSR and green innovation.

Regarding the relationships between CSR to external and internal stakeholders, and business sustainability performance, GIC were found to mediate the relationships between CSR to external stakeholders and ECP, SOP, and ENP (H_10-12_). These findings are consistent with those of Yuan and Cao [20] who investigated GDC. Specifically, the findings align with those of Singh et al. [11], who found that stakeholder pressures affect an organization's GDC, affecting its capacity for green innovation and ultimately influencing its overall performance. However, the study also found that GIC do not mediate the relationship between CSR to internal stakeholders and ECP, SOP, and ENP (H_14-16_). These results are inconsistent with those of Makkonen et al. [88] and Yuan and Cao [20] who integrated GIC as mediator. Furthermore, GIC significantly influenced the relationships between GDC and ECP, SOP, and ENP (H_17-19_). As a result, we argue that GDC is very important for improving ECP, SOP, and ENP, in the presence of GIC.

### 5.1 Theoretical contribution

This study makes significant theoretical contributions to several important areas. In particular, the incorporation of the DCV theory into the specific context of Chinese organizations represents a significant step forward. It highlights the adaptability and universality of dynamic capabilities across diverse cultural and business environments and underscores their critical role in shaping an organization's capacity to react to external influences. This theoretical framework provides a robust tool for examining how Chinese organizations respond to external forces, particularly in the context of CSR practices. This framework serves as a roadmap for achieving sustainable business performance in a sustainable manner, creating a win-win situation for both firms and their stakeholders. In addition, this study contributes to advancing the existing literature on CSR to external and internal stakeholders, GDC, GIC, ECP, SOP, and ENP in the context of local and international manufacturing firms in China. The existing literature lacks a comprehensive perspective on CSR practices, green innovation, and sustainable business performance in China.

Moreover, this study advances the theoretical discourse on stakeholder influence by examining the complex interplay between various stakeholder groups encompassing external and internal stakeholders. By adopting a multidimensional approach, our research deepens the understanding of stakeholder management and broadens the scope of stakeholder theory by revealing the intricate dynamics of stakeholder engagement and its impact on organizational strategies and outcomes.

Another notable contribution derives from the integration of DCV theory into CSR practices and the green innovation of local and international medium- and large-sized manufacturing firms in China. This builds on previous research that employed stakeholder, motivational, and contingency theories [72] to understand the impact of CSR practices on organizations’ green innovation and sustainability performance. Furthermore, integrating the two mediators–GDC and GIC–strengthens the relationship between stakeholders and sustainable business performance. While prior research has utilized a limited perspective in which only one of these variables has been examined, this study departs from this norm by employing both GDC and GIC as mediators. Previous studies have explored the impact of green social capital [14] and GDC [20] on sustainable business performance; however, none have examined the interplay between these variables in the context of mediation. Finally, our focus on the economic, environmental, and social dimensions of sustainable performance highlights the comprehensive and holistic nature of sustainability. This approach is consistent with the latest developments in sustainability reporting and recognizes the interconnectedness of various performance aspects. Moreover, it contributes significantly to the ongoing theoretical discourse by recognizing sustainability as a multi-dimensional construct.

### 5.2 Managerial implications

This study has practical implications for managers when making informed decisions. The results offer a framework that enables professionals to understand how business sustainability can be achieved in highly competitive markets. The findings suggest that firms must consider both external and internal stakeholders in their business operations to gain a competitive advantage. Managers can utilize stakeholder engagement strategies to address the specific requirements and anticipation of various stakeholder groups by acknowledging the diverse impacts of external and internal stakeholders, and organizations can implement more efficient and focused CSR initiatives. To align the interests of stakeholders and organizations, firms must produce their products in a manner that does not negatively affect stakeholders while still improving sustainable business performance from an economic perspective.

Additionally, managers may utilize the results to acknowledge the pivotal function of dynamic capabilities in navigating external pressures. Organizations may allocate resources towards cultivating and improving these dynamic capabilities to attain sustainability objectives, allowing them to adjust, innovate, and respond appropriately to stakeholder demands. Moreover, this study emphasizes the critical importance of GIC in the pursuit of sustainability. Managers should prioritize innovation in their products, processes, and systems to minimize environmental impact and optimize sustainability performance. This involves the cultivation of a culture of ongoing improvement and environmental accountability.

The findings of this study have significant practical implications for organizations seeking to create social value in the market and build solid relationships with stakeholders. Specifically, it highlights the crucial roles of GDC and GIC as mediators that impact social performance and eventually build trust between firms and stakeholders. Managers must prioritize adapting their resources to the changing business environment and create a growing demand for eco-friendly business practices among stakeholders.

This study underscores the significance of considering the long-term effects of CSR practices. Managers should adopt a proactive approach that aligns CSR initiatives with the organization's long-term objectives while also considering the economic, environmental, and social dimensions of sustainability. Thus, managers should promote interdisciplinary collaboration within their organizations by encouraging the cooperation of different departments toward the attainment of sustainability goals.

### 5.3 Limitations and future directions

This study had certain limitations that are worth noting. One limitation is the industry-specific nature of the sample, which is confined to manufacturing firms. Therefore, the findings of this study cannot be generalized to other industries. However, this limitation provides an opportunity for future researchers to extend the current study to other contexts. Another limitation is its cross-sectional design, which captures a snapshot of the relationships at a specific point in time. A longitudinal approach could provide insight into how these relationships evolve over time. Another limitation of this study is its geographical scope, which was limited to China. Thus, the results are not applicable to other manufacturing firms in different countries because of China's unique geographical, political, cultural, and social structures. Thus, future cross-country studies should be conducted to broaden the scope of these findings.

In addition, this study employs a quantitative methodology that can be complemented by qualitative insights. Integrating quantitative and qualitative data can provide a more comprehensive and in-depth understanding of the intricate relationships between CSR practices, dynamic capabilities, green innovation, and sustainability outcomes. Furthermore, while our study does not test the direct relationships between CSR and the three performance measures, we recognize the value in exploring this area further. Future research could investigate these direct links to provide a more comprehensive understanding of the interplay between CSR and firm performance. Moreover, this study could be expanded by including other important constructs, such as supply chain management and some of the 17 sustainable development goals (SDGs) that were not included in the current setting. This will allow future researchers to align their research with SGDs in China and contribute to ongoing efforts towards sustainable development. Further, expanding research to examine micro-(e.g., individual behaviors) and macro-level factors (e.g., government policies and global initiatives) can offer a more comprehensive understanding of sustainability dynamics. Finally, as this study integrated validated measures of sustainability from the existing literature, future studies should incorporate established Chinese indices of sustainability to assess stakeholder orientation.

## 6 Conclusions

This study focuses on modeling the significance of CSR practices on green innovation and sustainability performance. Specifically, we developed a model based on DCV theory to examine the impact of stakeholders on organizations' economic, environmental, and social performance, mediated by GDC and GIC. The research model is tested using data collected from 634 manufacturing firms. We proposed eight direct hypotheses, of which 7 were validated and one was rejected. Meanwhile, of the 17 indirect hypotheses, 11 were supported, and six were rejected. The findings demonstrate that stakeholder pressure affects both GDC and GIC, which subsequently impact the organization's sustainable performance in terms of economic, environmental, and social aspects. Moreover, firm-specific moderators, including firm size, age, origin, and cross-border e-commerce engagement, introduce intriguing nuances into these dynamics and shed light on the distinct impacts of these variables.

This study adds to the existing body of knowledge in business, sustainability, and stakeholder management domains. It offers both theoretical and practical guidance to organizations seeking to improve their sustainability performance, respond to stakeholder expectations, and navigate complex business landscapes. As global interest in sustainability continues to grow, this study provides a comprehensive framework for organizations to develop and enhance their capabilities, foster innovation, and advance sustainability across economic, environmental, and social dimensions, thus positioning them for long-term success in an ever-changing world.

## Ethics approval

The Human Research Ethics Committee of Business School, Nantong Institute of Technology, China approved this study (BS-NIT-2023-0417). This study has been performed in accordance with the Declaration of Helsinki.

## Informed consent

Written informed consent for participation was obtained from respondents who participated in the survey.

## Consent to publish

All authors approved the manuscript and give their consent for submission and publication.

## Availability of data and materials

The original contributions presented in the study are included in the article/Supporting Material (**S3. Dataset)**, further inquiries can be directed to the corresponding author/s.

## Funding

This study is supported via funding from 10.13039/501100002949Jiangsu Province "14th Five-Year Plan" Business Administration Key Construction Discipline Project (Su Jiaoyanhan [2022] No. 2/Sequence 285) and Xinyingyun E-commerce Operation Service (Project No.: XQPT003).

## CRediT authorship contribution statement

**Han Xiao:** Writing – original draft, Methodology, Conceptualization. **Abdullah Al Mamun:** Writing – review & editing, Methodology, Formal analysis, Conceptualization. **Mohammad Nurul Hassan Reza:** Writing – original draft, Methodology, Conceptualization. **Xiaofang Lin:** Writing – original draft, Methodology, Conceptualization. **Qing Yang:** Writing – review & editing, Methodology, Formal analysis, Conceptualization.

## Declaration of competing interest

The authors declare the following financial interests/personal relationships which may be considered as potential competing interests:Abdullah Al Mamun reports financial support was provided by JIangsu Education Department. Corresponding author (Abdullah Al Mamun) is the Associate Editor of the Journal "Heliyon" If there are other authors, they declare that they have no known competing financial interests or personal relationships that could have appeared to influence the work reported in this paper.
