# Supplementary material for: Modeling the significance of corporate social responsibility on green capabilities and sustainability performance
Source: Heliyon. 2024 Oct 5;10(19):e38991. doi: 10.1016/j.heliyon.2024.e38991 (PMC11492346; doi:10.1016/j.heliyon.2024.e38991)
Supplement: Multimedia component 1 [file mmc1.docx]

**Supporting Material 1.** Survey Questionnaire

| CES1 | Our firm contributes to campaigns and projects that promote the well-being of the society |
| --- | --- |
| CES2 | Our firm implements special programs to minimize its negative impact on the natural environment. |
| CES3 | Our company participates in activities which aim to protect and improve the quality of the natural environment. |
| CES4 | Our firm targets sustainable growth which considers future generations. |
| CES5 | Our firm makes investment to create a better life for future generations. |
| CES6 | Our firm encourages its employees to participate in voluntarily activities |
| CES7 | Our firm supports nongovernmental organizations working in problematic areas. |
| CIS1 | Our firm supports employees who want to acquire additional education. |
| CIS2 | Our firm policies encourage the employees to develop their skills and careers. |
| CIS3 | Our firm implements flexible policies to provide a good work & life balance for its employees. |
| CIS4 | The management of our company is primarily concerned with employees’ needs and wants. |
| CIS5 | The managerial decisions related with the employees are usually fair. |
| CIS6 | Our firm provides full and accurate information about its products. |
| CIS7 | Our firm respects consumer rights beyond the legal requirements. |
| CIS8 | Customer satisfaction is highly important for our company. |
| CIS9 | Our firm complies with legal regulations completely and promptly. |
| GIC1 | We frequently adopt new skills to develop novel green products to simplify their production and packaging. |
| GIC2 | We frequently adopt new skills to develop novel green products to minimize damage from waste. |
| GIC3 | We frequently adopt the latest production techniques to reduce waste. |
| GIC4 | We frequently update procedures to effectively reduce emissions of hazardous substances or waste. |
| GIC5 | We frequently develop new tactics to update all relevant stakeholders on issues concerning green innovation during decision-making. |
| GIC6 | We frequently develop new approaches to fit green innovation into management and administration to improve performance. |
| GDC1 | Our firm has the ability to quickly monitor the environment to identify new green opportunities. |
| GDC2 | Our firm has the ability to assimilate, learn, generate, combine, share, transform, and apply new green knowledge. |
| GDC3 | Our firm has the ability to successfully integrate and manage specialized green knowledge within the company. |
| GDC4 | Our firm has the ability to successfully coordinate employees to develop green technology. |
| GDC5 | Our firm has the ability to successfully allocate resources to develop green innovations. |
| ECP1 | Our revenue has grown steadily over the last five years. |
| ECP2 | Our profits have grown steadily over the last five years. |
| ECP3 | Our customer’s base has grown steadily over the last five years. |
| ECP4 | Our market share grows steadily over the last five years. |
| ECP5 | The number of new environmentally friendly products has increased steadily over the last five years. |
| ECP6 | Our environmental performance has increased over the last five years. |
| SOP1 | Our firm provides policy protection against the discrimination of stakeholders. |
| SOP2 | Our firm provides training on cultural diversity to employees. |
| SOP3 | Our firm provides equipment to employ people with disabilities. |
| SOP4 | Abuse of any kind toward stakeholders is not tolerated. |
| SOP5 | Our firm promotes the involvement of employees in community support programs. |
| SOP6 | Our firm promotes charitable initiatives. |
| ENP1 | Our firm has achieved important environment-related certifications (e.g., ISO 14000). |
| ENP2 | Our firm has regularly achieved targets imposed on energy conservation, recycling, and waste reduction. |
| ENP3 | Due to its environment-friendly practices, our firm has saved a significant amount of money in the past. |
| ENP4 | On average, the overall environmental performance of my company has improved in the past five years. |
| ENP5 | Our firm has adopted process management which includes pollution control and waste emissions. |

**Note:** CES - CSR to External Stakeholders; CIS - CSR to Internal Stakeholders; GDC - Green Dynamic Capability; GIC - Green Innovation Capability; ECP - Economic Performance; SOP - Social Performance; ENP - Environmental Performance
