# Supplementary material for: Modeling the significance of corporate social responsibility on green capabilities and sustainability performance
Source: Heliyon. 2024 Oct 5;10(19):e38991. doi: 10.1016/j.heliyon.2024.e38991 (PMC11492346; doi:10.1016/j.heliyon.2024.e38991)
Supplement: Multimedia component 2 [file mmc2.docx]

**Supporting Material 2.** Loading, Cross-Loading and Fornell-Larcker criterion

|  | CES | CIS | GDC | GIC | ECP | SOP | ENP |
| --- | --- | --- | --- | --- | --- | --- | --- |
| CES1 | 0.749 | 0.372 | 0.097 | 0.157 | 0.218 | 0.324 | 0.262 |
| CES2 | 0.777 | 0.327 | 0.122 | 0.135 | 0.238 | 0.368 | 0.319 |
| CES3 | 0.774 | 0.336 | 0.152 | 0.144 | 0.261 | 0.340 | 0.318 |
| CES4 | 0.849 | 0.362 | 0.150 | 0.189 | 0.287 | 0.361 | 0.371 |
| CES5 | 0.823 | 0.354 | 0.146 | 0.164 | 0.225 | 0.346 | 0.285 |
| CES6 | 0.868 | 0.376 | 0.218 | 0.278 | 0.276 | 0.372 | 0.371 |
| CES7 | 0.798 | 0.346 | 0.176 | 0.202 | 0.165 | 0.277 | 0.291 |
| CIS1 | 0.292 | 0.720 | 0.113 | 0.142 | 0.362 | 0.346 | 0.254 |
| CIS2 | 0.271 | 0.688 | 0.061 | 0.099 | 0.395 | 0.353 | 0.251 |
| CIS3 | 0.341 | 0.690 | 0.012 | 0.093 | 0.360 | 0.329 | 0.266 |
| CIS4 | 0.333 | 0.818 | 0.131 | 0.157 | 0.440 | 0.399 | 0.300 |
| CIS5 | 0.378 | 0.781 | 0.129 | 0.143 | 0.413 | 0.396 | 0.262 |
| CIS6 | 0.373 | 0.774 | 0.138 | 0.143 | 0.358 | 0.363 | 0.303 |
| CIS7 | 0.320 | 0.778 | 0.161 | 0.195 | 0.352 | 0.298 | 0.313 |
| CIS8 | 0.350 | 0.749 | 0.081 | 0.105 | 0.332 | 0.355 | 0.293 |
| CIS9 | 0.326 | 0.780 | 0.103 | 0.161 | 0.406 | 0.369 | 0.284 |
| GDC1 | 0.181 | 0.128 | 0.925 | 0.651 | 0.118 | 0.156 | 0.198 |
| GDC2 | 0.137 | 0.117 | 0.906 | 0.650 | 0.165 | 0.140 | 0.248 |
| GDC3 | 0.184 | 0.172 | 0.895 | 0.595 | 0.201 | 0.208 | 0.271 |
| GDC4 | 0.208 | 0.158 | 0.901 | 0.624 | 0.132 | 0.151 | 0.226 |
| GDC5 | 0.184 | 0.116 | 0.924 | 0.682 | 0.129 | 0.152 | 0.215 |
| GIC1 | 0.223 | 0.182 | 0.616 | 0.895 | 0.166 | 0.203 | 0.256 |
| GIC2 | 0.221 | 0.154 | 0.580 | 0.879 | 0.158 | 0.224 | 0.239 |
| GIC3 | 0.172 | 0.140 | 0.581 | 0.824 | 0.158 | 0.162 | 0.199 |
| GIC4 | 0.205 | 0.185 | 0.663 | 0.886 | 0.156 | 0.202 | 0.266 |
| GIC5 | 0.165 | 0.154 | 0.607 | 0.845 | 0.182 | 0.206 | 0.226 |
| GIC6 | 0.237 | 0.175 | 0.601 | 0.852 | 0.124 | 0.179 | 0.197 |
| ECP1 | 0.275 | 0.398 | 0.141 | 0.175 | 0.793 | 0.427 | 0.317 |
| ECP2 | 0.211 | 0.351 | 0.114 | 0.090 | 0.784 | 0.372 | 0.301 |
| ECP3 | 0.295 | 0.445 | 0.102 | 0.127 | 0.788 | 0.452 | 0.297 |
| ECP4 | 0.203 | 0.401 | 0.161 | 0.130 | 0.777 | 0.319 | 0.271 |
| ECP5 | 0.209 | 0.396 | 0.134 | 0.132 | 0.789 | 0.438 | 0.330 |
| ECP6 | 0.186 | 0.351 | 0.107 | 0.165 | 0.758 | 0.311 | 0.256 |
| SOP1 | 0.339 | 0.388 | 0.105 | 0.129 | 0.400 | 0.780 | 0.384 |
| SOP2 | 0.347 | 0.395 | 0.145 | 0.188 | 0.417 | 0.821 | 0.396 |
| SOP3 | 0.293 | 0.371 | 0.107 | 0.162 | 0.379 | 0.747 | 0.417 |
| SOP4 | 0.349 | 0.390 | 0.163 | 0.219 | 0.409 | 0.832 | 0.390 |
| SOP5 | 0.362 | 0.341 | 0.136 | 0.184 | 0.378 | 0.776 | 0.365 |
| SOP6 | 0.283 | 0.319 | 0.162 | 0.163 | 0.343 | 0.743 | 0.299 |
| ENP1 | 0.371 | 0.353 | 0.204 | 0.250 | 0.339 | 0.433 | 0.834 |
| ENP2 | 0.264 | 0.302 | 0.189 | 0.175 | 0.253 | 0.317 | 0.754 |
| ENP3 | 0.344 | 0.303 | 0.224 | 0.228 | 0.328 | 0.431 | 0.845 |
| ENP4 | 0.311 | 0.315 | 0.205 | 0.216 | 0.340 | 0.387 | 0.826 |
| ENP5 | 0.308 | 0.240 | 0.209 | 0.212 | 0.262 | 0.359 | 0.806 |
| *Fornell-Larcker criterion* | | | | | | | |
| CES | 0.806 |  |  |  |  |  |  |
| CIS | 0.437 | 0.754 |  |  |  |  |  |
| GDC | 0.196 | 0.151 | 0.910 |  |  |  |  |
| GIC | 0.236 | 0.192 | 0.704 | 0.864 |  |  |  |
| ECP | 0.296 | 0.500 | 0.163 | 0.182 | 0.782 |  |  |
| SOP | 0.420 | 0.468 | 0.177 | 0.227 | 0.494 | 0.784 |  |
| ENP | 0.396 | 0.373 | 0.254 | 0.268 | 0.377 | 0.478 | 0.814 |

**Note:** CES - CSR to External Stakeholders; CIS - CSR to Internal Stakeholders; GDC - Green Dynamic Capability; GIC - Green Innovation Capability; ECP - Economic Performance; SOP - Social Performance; ENP - Environmental Performance
